# Screening for germline *BRCA1*, *BRCA2*, *TP53* and *CHEK2* mutations in families at-risk for hereditary breast cancer identified in a population-based study from Southern Brazil

**DOI:** 10.1590/1678-4685-GMB-2014-0363

**Published:** 2016-05-24

**Authors:** Edenir Inêz Palmero, Bárbara Alemar, Lavínia Schüler-Faccini, Pierre Hainaut, Carlos Alberto Moreira-Filho, Ingrid Petroni Ewald, Patricia Koehler dos Santos, Patricia Lisbôa Izetti Ribeiro, Cristina Brinkmann de Oliveira, Florence Le Calvez Kelm, Sean Tavtigian, Silvia Liliana Cossio, Roberto Giugliani, Maira Caleffi, Patricia Ashton-Prolla

**Affiliations:** 1 Programa de Pós Graduação em Genética e Biologia Molecular, Universidade Federal do Rio Grande do Sul, Porto Alegre, RS, Brazil; 2 Laboratório de Medicina Genômica, Hospital de Clinicas de Porto Alegre, Porto Alegre, RS, Brazil; 3 Cluster of Molecular Carcinogenesis, International Agency for Research on Cancer, Lyon, France; 4 Centro de Pesquisa em Oncologia Molecular, Hospital de Câncer de Barretos, Barretos, SP, Brazil; 5 Faculdade de Ciências da Saúde Dr. Paulo Prata, São Paulo, SP, Brazil; 6 Serviço de Genética Médica, Hospital de Clinicas de Porto Alegre, Porto Alegre, RS, Brazil; 7 Departmento de Genética, Universidade Federal do Rio Grande do Sul, Porto Alegre, RS, Brazil; 8 Centro de Pesquisa Experimental, Instituto de Educação e Pesquisa Albert Einstein, São Paulo, SP, Brazil; 9 Departmento de Imunologia, Instituto de Ciências Biomédicas, Universidade de São Paulo, São Paulo, SP, Brazil; 10 Programa de Pós Graduação em Gastroenterologia, Universidade Federal do Rio Grande do Sul, Porto Alegre, RS, Brazil; 11 Nucleo Mama Porto Alegre e Associação Hospitalar Moinhos de Vento, Porto Alegre, RS, Brazil

**Keywords:** Breast cancer predisposition syndrome, hereditary breast cancer, genetic cancer risk assessment

## Abstract

In Brazil, breast cancer is a public health care problem due to its high incidence and mortality rates. In this study, we investigated the prevalence of hereditary breast cancer syndromes (HBCS) in a population-based cohort in Brazils southernmost capital, Porto Alegre. All participants answered a questionnaire about family history (FH) of breast, ovarian and colorectal cancer and those with a positive FH were invited for genetic cancer risk assessment (GCRA). If pedigree analysis was suggestive of HBCS, genetic testing of the *BRCA1, BRCA2, TP53*, and *CHEK2* genes was offered. Of 902 women submitted to GCRA, 214 had pedigrees suggestive of HBCS. Fifty of them underwent genetic testing: 18 and 40 for *BRCA1*/*BRCA2* and *TP53* mutation screening, respectively, and 7 for *CHEK2* 1100delC testing. A deleterious *BRCA2* mutation was identified in one of the HBOC probands and the *CHEK2* 1100delC mutation occurred in one of the HBCC families. No deleterious germline alterations were identified in *BRCA1* or *TP53*. Although strict inclusion criteria and a comprehensive testing approach were used, the suspected genetic risk in these families remains unexplained. Further studies in a larger cohort are necessary to better understand the genetic component of hereditary breast cancer in Southern Brazil.

## Introduction

Breast cancer (BC) is a significant health care problem worldwide, and approximately 1.67 million new cases were diagnosed in 2012, representing 25% of all cancers (Globocan). In Latin America, BC is the most prevalent solid tumor diagnosed in women in the majority of countries (Goss *et al.*, 2013). In Brazil, 51,120 new BC diagnoses were estimated for 2014, and the disease is the first cause of death by cancer in women of all ages, especially in young women, under the age of 50 years (DataSUS, 2014; INCa, 2014). The South and Southeast regions of Brazil have the highest BC incidence rates: 70.98 and 71.98 cases in 100,000 women respectively (INCa, 2014). In comparison to national figures, Rio Grande do Sul, the southernmost State, presents high BC incidence (87.12:100,000) and mortality rates (13.18:100,000), adjusted rates in 2011 (INCa, 2014).

An estimated 5-10% of all BCs are hereditary, *i.e.* caused by germline mutations in high-penetrance cancer predisposition genes (King *et al.*, 2003). Of these, the more prevalent mutations are in *BRCA1* (OMIM#113705) and *BRCA2* (OMIM#600185) (Miki *et al.*, 1994; Wooster *et al.*, 1994), tumor suppressor genes which are associated with hereditary breast and ovarian cancer (HBOC) syndrome. Lifetime risks of breast and ovarian cancer are as high as 65-85% in *BRCA1* and 45-85% in *BRCA2* mutation carriers (Antoniou *et al.*, 2006; Cass *et al.*, 2003; Euhus and Robinson, 2013). To date, over 3.000 distinct germline mutations, polymorphisms and sequence variants have been described in *BRCA1* and *BRCA2*, spread throughout both genes (Breast Cancer Information Core, BIC, 2014). Most are point mutations, small insertions or deletions. However, large genomic deletions and duplications involving one or more exons of *BRCA1*, and less commonly, *BRCA2*, have been reported (Gutiérrez-Enríquez *et al.*, 2007; Preisler-Adams *et al.*, 2006; Thomassen *et al.*, 2006). Most of these mutations are caused by recombination events involving Alu repeats that are particularly numerous in the *BRCA1* locus (Payne *et al.*, 2000). The proportion of genomic rearrangements over all *BRCA1* gene mutations in HBOC families seems to be population-dependent varying from 2% in a series of American families (Hendrickson *et al.*, 2005) to 36% in Dutch patients (Petrij-Bosch *et al.*, 1997). In Rio de Janeiro, (Moreira *et al.*, 2012) screened 168 BC affected women for *BRCA2* c.156_157insAlu and found 3 unrelated carriers. Other studies have screened cohorts of Brazilian BC patients for specific mutations, or have focused on some subgroups (for example young women) (Carraro *et al.*, 2013; Ewald *et al.*, 2011; Gomes *et al.*, 2007) but the exact prevalence of any *BRCA* mutation remains largely unknown.

Besides *BRCA1* and *BRCA2*, inherited mutations in other tumor suppressor genes also increase the risk for breast cancer and other tumors. Highly penetrant, hereditary breast cancer genes include *PTEN* (Cowden's syndrome), *TP53* (Li-Fraumeni syndrome), *STK11* (Peutz-Jeghers syndrome) and *CDH1* (Hereditary diffuse gastric cancer. Germline mutations in *CHEK2* gene are associated with a modest increase in the risk of breast (15-25% lifetime risk) and colorectal cancer in the hereditary breast and colon cancer syndrome (HBCC) (Euhus and Robinson, 2013; Meijers-Heijboer *et al.*, 2003).

In this study, we analyzed a population-based cohort of women recruited from primary health care units in Porto Alegre who were referred to genetic cancer risk assessment (GCRA) whenever they had a positive family history for breast, ovarian and colorectal cancer (Palmero *et al.*, 2009). Those women with pedigrees suggestive of a hereditary breast cancer predisposition syndrome were offered genetic testing for germline mutations in one or more of the main breast cancer predisposition genes (*BRCA1, BRCA2, TP53*, *PTEN* and *CHEK2*). Our aim was to evaluate the prevalence of hereditary breast cancer phenotypes and breast cancer predisposition gene variations among at-risk women recruited from a population-based cohort in a region with high breast cancer incidence.

## Subjects and Methods

### Patient Recruitment

In 2004, a large population-based cohort study (the Núcleo Mama Porto Alegre – NMPOA-Cohort) was started in Porto Alegre, Southern Brazil, through a partnership between government, non-profit community-based organizations, universities and private entities. This prospective cohort intends to collect demographic and epidemiological data of a large sample of women and test a model for community- based breast cancer screening in an underserved population (Caleffi *et al.*, 2010) (Smith *et al.*, 2006) in an attempt to ultimately decrease BC mortality rates in this region. The study recruited women above the age of 15 years who sought health care in 18 primary health care units (PCUs) located in specific city regions within a 24-month period. Family history (FH) of breast, ovarian and colorectal cancer was assessed in women (above age 15 years) seen in 18 primary health care units from the region by a brief questionnaire and is the basis for the study described elsewhere as the Genetic Cancer Risk Assessment Program of the NMPOA cohort (Palmero *et al.*, 2009). The seven questions of this instrument refer to family history features that have been associated with an increased likelihood of clinically significant *BRCA* mutations and thus, these questions were designed primarily to identify patients at-risk for HBOC syndrome (Couch *et al.*, 1997; Frank *et al.*, 2002; Nelson *et al.*, 2005; Shattuck-Eidens *et al.*, 1997; Srivastava *et al.*, 2001). In addition, a question about family history (FH) of breast and/or colon cancer was included due to a previous suggestion of a higher than expected number of families with these tumors in cancer genetic clinics of Porto Alegre (Palmero *et al.*, 2007). The questionnaire is presented in Table S1. Patients answering positively to at least one of the seven questions in the primary health care unit were referred to genetic risk assessment at NMPOA.

Genetic evaluation included medical and family histories recorded in detailed pedigrees with information traced as far backwards and laterally as possible, extending to paternal lines and including a minimum of three generations. Confirmation of the cancer FH was attempted in all cases and pathology and medical records, as well as death certificates, were obtained whenever possible upon specific consent from the patient and/or her family. Lifetime breast cancer risk estimates were obtained using the Claus, Gail, and Tyrer-Cuzick models. All pedigrees were reviewed by at least two clinical geneticists to assess presence of criteria for the diagnosis of LFS, LFL, HBCC, Cowden or other cancer predisposition syndromes. Patients fulfilling criteria for a breast cancer predisposition syndrome were candidates for the present study and offered genetic testing. For the clinical diagnosis of HBOC syndrome, the National Comprehensive Cancer Network (NCCN) and American Society of Clinical Oncology (ASCO) criteria were used (Ford *et al.*, 1994) (NCCN, 2014). In addition, prior probabilities of carrying a *BRCA1* or *BRCA2* mutations were determined for each patient using mutation prevalence tables and the modified Couch mutation prediction model (Domchek *et al.*, 2003; Frank *et al.*, 2002). For LFS, the original criteria described by Li and Fraumeni (Li *et al.*, 1988) were used; for LFL, pedigrees were classified according to the criteria of Birch (Birch *et al.*, 1994) and Eeles (Eeles, 1995); for HBCC and Cowden's syndrome, the criteria described by (Meijers-Heijboer *et al.*, 2003) and (Nelen *et al.*, 1996), respectively, were used. Criteria to indicate genetic testing for HBOC were set so as not to miss any of the families at high risk for germline *BRCA1* and *BRCA2* mutations and included families fulfilling the ASCO criteria and/or who had a prior probability of mutation in a *BRCA* gene of 30% or more (Domchek *et al.*, 2003; Frank *et al.*, 2002). Genetic testing included mutation analysis of one or more of the four main breast cancer predisposition genes (*BRCA1*, *BRCA2*, *TP53*, and *CHEK2*). Initially, the index case was approached and informed of the study. Invitation to participate was made directly to all cancer-affected index-cases. In those unaffected by cancer an attempt was made to invite at-risk, cancer affected relatives. Blood samples were obtained from cancer-affected women and/or their family members after informed consent, depending on accessibility of individuals and willingness to participate in the study. The study was approved by the Institutional Review Board of the participating centers.

### Screening for germline mutations

DNA samples were obtained from peripheral blood, using a commercial DNA extraction kit, Gentra Puregene Blood kit, according manufacturer's instructions (Qiagen, Valencia, CA, USA). All DNA samples were screened for *BRCA1/2* and *TP53* mutations using Denaturing High Performance Liquid Chromatography (DHPLC) or High Resolution Melting (HRM), and samples showing variants were submitted to Sanger sequencing.

#### BRCA1/BRCA2 genes

All samples were amplified by PCR reactions using oligonucleotide primers and corresponding annealing temperatures previously described in the literature (Friedman *et al.*, 1997; Friedman *et al.*, 1994). Mutation screening by DHPLC was carried out using a WAVE MD 4000 DNA Fragment Analysis System equipped with a DNASep Cartridge (Transgenomic Inc., Omaha NE, USA) as described elsewhere (Fackenthal *et al.*, 2005). The HRM curve analysis was performed in a LightScanner instrument (Idaho Tecnology Inc.) using the Light Scanner Mastermix with LCGreen dye (Idaho Tecnology Inc.). Heterozygous profiles were identified by visual inspection of the chromatograms/melting curves and putative sequence variants were re-analyzed by bi-directional sequencing on a MegaBACE 1000 (Amersham Biosciences, Buckinghamshire, UK) or an ABI-PRISM 3100 Genetic Analyzer (Applied Biosystems, Foster City, USA) using an independent PCR product. Sequence alterations were classified based on data available in the Breast Cancer Information Core (BIC, 2014), ClinVar (Landrum *et al.*, 2014), Universal Mutation database - UMD (Caputo *et al.*, 2012) and AlignGVGD (Tavtigian *et al.*, 2006). New or pathogenic mutations were also searched in The Human Gene Mutation Database (HGMD, 2014,) and LOVD (Vallée *et al.*, 2012). All the 18 HBOC families were screened for *BRCA1* genomic rearrangements and 10 of these families were screened for *BRCA2* genomic rearrangements using Multiplex Ligation-dependent Probe Amplification (MLPA) methodology using the SALSA P002B *BRCA1* and SALSA P045 *BRCA2* MLPA probe mix assays (MRC-Holland, Amsterdam, The Netherlands) as recommended by the manufacturer (MRC-Holland) and information on copy number was extracted with Coffalyser V9.4 Software (MRC-Holland). All analyses were performed in duplicate and in at least two independent experiments.

#### TP53 gene

Patients fulfilling Li-Fraumeni and Li-Fraumeni-like syndrome criteria (Birch *et al.*, 1994; Eeles, 1995; Li *et al.*, 1988) were screened for *TP53* germline mutations as follows: exons 2-11 were screened by HRM (as described by (Garritano *et al.*, 2009) followed by bi-directional sequencing of altered regions. Sequence variations were classified according to data submitted to the *TP53* database at the International Agency for Research on Cancer - IARC version R17 (Petitjean *et al.*, 2007). All possible deleterious mutations were confirmed by a second and independent analysis.

#### CHEK2 gene

Families with a history of breast and colorectal cancer consistent with HBCC syndrome were screened for a specific *CHEK2* mutation (1100delC), located in exon 10 by PCR amplification followed by direct sequencing. To ensure amplification of the functional copy of *CHEK2* and exclusion of *CHEK2* pseudogenes, a strategy of long-range PCR amplifications with primers designed outside the pseudogene sequences was used as described by (Vahteristo *et al.*, 2001).

## Results

### Sample characteristics

Of all 9,234 women included in the NMPOA cohort (Porto Alegre, Brazil), 1,286 (13.9%) answered positively to at least one of the seven questions about FH. Those above 18 years (n = 1,247) were invited for GCRA. Of the 1,247 patients referred to GCRA, 902 (72.3%) effectively participated in the assessment and of these, 214 (23.7%) women from 183 families fulfilled criteria for one or more of the breast cancer predisposition syndromes (BCPS) considered in our study: 65 fulfilled criteria for HBOC, 122 for LFL and 22 for HBCC syndromes.

None of the patients assessed reported a personal and/or family history suggestive of classic Li-Fraumeni and Cowden's Syndrome. Detailed information on study design, patient recruitment, and demographic data of the 902 patients seen for GCRA is described elsewhere (Palmero *et al.*, 2009).

Of the 214 women with criteria for a BCPS, 64 (29.9%; corresponding to 50 families) decided to continue with the genetic investigation and proceeded to germline mutation testing. An additional 54 cancer-unaffected and at-risk patients (25.2%) attempted contact with their cancer-affected relatives to invite them for GCRA but they did not schedule an appointment. Among the 50 probands tested, the most frequent cancer site was breast, and as expected, the majority of these diagnoses were made before the age of 50 years. Only two probands were diagnosed with ovarian cancer (one HBOC and one LFL family). Moreover, six probands had colorectal cancer (3 LFL and 3 HBCC families). Ten of the 50 probands were cancer unaffected (from 7 LFL families and three from families with both HBOC and LFL criteria). However, four of them were supposedly obligate carriers by family history. Additional information on cancer site and phenotype among the 50 families studied is summarized in Tables 1 and 2. At least one tumor diagnosis was confirmed by pathology reports, medical records, or death certificates in 45/50 (90%) families. However, confirmation of a sufficient number of cancer diagnoses to affirm with certainty the BCPS phenotype was only possible in 13 families (26%). Due to the high frequency of breast cancer diagnoses in the 50 families studied, 14 (28%) of them fulfilled criteria for more than one BCPS when testing was indicated and therefore, these families were screened for germline mutations in more than one predisposition gene. Thus, eight families were tested for *BRCA1/BRCA2* and *TP53* germline mutations, two families for *BRCA1/BRCA2* and *CHEK2* mutations, three families for *TP53* and *CHEK2* mutations, and one family was screened for mutations in all four genes (*BRCA1/BRCA2*, *CHEK2* and *TP53*). During the process of genetic testing, three families presented additional information of the presumed cancer diagnoses and one of the phenotypes was excluded (HBOC in family 681, HBCC in family 284, and LFL in two families, 163 and 186) (Table 2).

**Table 1:** Cancer phenotype of the 50 BCPS families submitted to genetic testing.

BCPS Criteria	Number of families*	Average age at BC dx (years)	Number of cancer diagnoses/family	Occurrence of Childhood tumors	Two or more tumors diagnosed < 45years	Number of cancer-affected generations
	N	Mean (± SD)	Mean (± SD)	N (%)	N (%)	Mean (± SD)
Criteria for one BCPS						
HBOC	18	45.2 (11.9)	6.7	2	47	2.7
(3.7)	(1.6)	(38.8)	(0.9)			
LFL	40	48.3	6.1	12	71	2.8
(12.9)	(3.3)	(4.9)	(28.9)	(0.9)		
HBCC	7	51.8 (15.6)	7.6	1	12	2.7
(1.8)	(1.9)	(22.6)	(0.5)			

(*) One family may fulfill more than one criterion. For a detailed description of the families with multiple criteria, refer to Table 2

**Table 2:** Detailed description of the 14 families that at inclusion were identified as having criteria for more than one BCPS.

Family ID	Criteria	Number of cancer diagnoses	Number of diagnoses confirmed **(** **1** **)**	Cancer-affected proband	Proband cancer site	Cancer diagnosed relatives (by site)
6	LFL (Birch/Eeles 1) + HBOC	7	2	Yes	**Colon (F-40)**	Breast (F-38, F-47, F-bil 39), **CNS** (F-38, **M-50**), lung (M-64)
25	LFL (Eeles 1) + HBCC	9	1	Yes	**Colon (F-72)**	Breast (F-60), colon (F-35, M-13), uterine (50), prostate (ND), pancreas (M-ND), esophageal (M- > 70)
101	LFL (Eeles 1/2) + HBOC	Mat = 3; Pat = 3	0	No	NA	Mat= breast (F-38, F-46), gastric (M-70);
						Pat= sarcoma (M-15), leukemia (M-49), gastric (F-ND)
103	LFL (Eeles 1) + HBCC	8	1	Yes	**Breast Bil (F-65)**	Breast (F-36, F-82), gynecologic (49), lung (F-ND), leukemia (M-ND), colon (M-ND), unknown (M-ND)
186 **(** **2** **)**	LFL (Birch) + HBOC	4	0	No	NA	Breast (F-35, F-40, F-40), kidney (M-5)
284 **(** **3** **)**	LFL (Eeles 1) + HBCC	5	0	Yes	Colon (F-71)	Breast (F-50), colon (F > 50), CNS (M > 70), gastric (M-41)
439	HBOC + HBCC	6	2	Yes	**Breast Bil (F < 50)**	**Breast** (**F-bil39**, F-70), uterine (> 30), colon (M-60), ovarian (70)
442	LFL (Eeles 1) + HBOC	18	1	Yes	**gastric (F-39),** ovarian (42)	Breast (F-15, F-40, F-21, F-ND, F-ND, F-ND, F-ND, F-ND, F-ND, F-ND), gastric (F-ND), prostate (ND), leukemia (M-48), kidney (F-ND), unknown (F-64, M-ND)
520	LFL (Birch) + HBOC	9	5	Yes	**Breast Bil (F-42)**	**Breast (F-49, F-38**, F < 52, **F bil < 55**), bone (M-31), **esophageal (M-50),** colon (F > 60), leukemia (M-8)
552 **(** **4** **)**	LFL (Eeles 1) + HBOC + HBCC	Mat = 10; Pat = 2	1	Yes	**Breast (F-38)**	Mat= Breast (F-36 F-62, F-25, F-ND), colon (M > 50, M < 48, M-ND), ovarian (ND), uterine (ND);
						Pat= brain (M-ND), prostate (ND)
554	LFL (Eeles 1) + HBOC	4	2	Yes	Breast (F-44),**melanoma (F-39)**	**Ovarian (63**, 60)
590	LFL (Eeles 1) + HBOC	8	3	Yes	**Breast (F-49)**	**Breast (F-53), thyroid (F-36),** ovarian (28), gastric (F-40), throat (M-50), nose (F-ND), unknown (F-ND)
681**(** **5** **)**	HBOC + HBCC	9	8	Yes	**Breast (F-52)**	**Lung (M-62), endometrial (64), uterine** (41, **< 50**), **breast (F < 50), colon (M-50, F-68), throat (M < 50)**
736	LFL (Eeles 1) + HBOC	10	0	No	NA	pancreas (F-25, M-50), uterine (32, 49), ovarian (32, 49, 36), colon (F-40), breast bil (F-47), lung (F-60)

1 Includes confirmation of cancer site and/or type by pathology reports, death certificates and/or review of medical records.

2 One of the cancer diagnoses was not confirmed, and therefore this family, although tested for *TP53* mutations, does not fulfill criteria for LFL (Birch) as thought initially

3 One of the cancer diagnoses was not confirmed, and therefore this family, although tested for the *CHEK2* 1100delC mutation, does not fulfill criteria for HBCC as thought initially

4 Family meets criteria for HBOC and HBCC in the maternal side and for LFL (Eeles1 and 2) in the paternal lineage of the proband.

5 One of the cancer diagnoses was not confirmed, and therefore this family, although tested for *BRCA1*/2 mutations, does not fulfill criteria for HBOC as thought initially

Mat= maternal side; Pat= Paternal side; Bil=bilateral; F=female; M=male; CNS= central nervous system; ND= not determined. Bold cancer cases means diagnosis confirmed.

### Mutation detection studies

#### HBOC

Nineteen individuals from 18 families with HBOC criteria underwent genetic testing. After DHPLC or HRM screening, the PCR products showing variant or dubious profiles were sequenced for confirmation. A total of 183 of 646 (28.3%) and 278 of 798 (34.8%) *BRCA1* and *BRCA2* amplicons were sequenced, respectively. There was a complete agreement between the results from DHPLC and sequencing for both *BRCA* genes.

**Figure 1:**
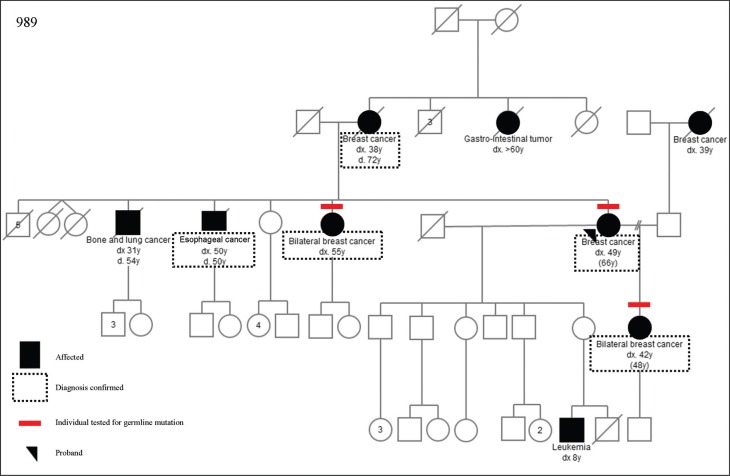
Pedigree of a family with p.E3002K mutation in *BRCA2* gene.

Sequencing results were compared to data deposited in the BIC, ClinVar, UMD, HGMD and LOVD databases. We identified 12 and 31 sequence variants in *BRCA1* and *BRCA2*, respectively (Table 3). Most of them were previously described and deposited in one or more databases as variants with no clinical significance. However, databases diverged about classification of some variants. In *BRCA1*, four variants were classified as variants of unknown significance (VUS) in at least one database, but none was classified as VUS in all three databases. In *BRCA2,* two variants were consistently classified as VUS in all databases: c.9004G > A (p.E3002K) and c.9581C > A (p.P3194Q). However, in HGMD the variant p.E3002K is described as a deleterious mutation, and its pathogenicity was demonstrated (Biswas *et al.*, 2012; Cote *et al.*, 2012; Karchin *et al.*, 2008). The pedigree from this family can be seen in Figure 1. We also found three new variants in *BRCA2*, not described in BIC, ClinVar, UMD, HGMD or LOVD: c.1402A > G (p.R468G), c.2842G > A (p.V948I) and c.7017G > A (p.K2339K). More detailed results can be found in Table 3. Screening for large gene rearrangements in all 18 HBOC probands for *BRCA1* and in 10 for *BRCA2* did not show any detectable abnormalities.

**Table 3:** *BRCA1* and *BRCA2* sequence variants identified in the 18 families fulfilling HBOC syndrome criteria.

Localization	Alteration*	Classification					
		UMD	BIC (Clinical importance)	ClinVar	Align-GVGD score***	Families with variant (N)	Detection method
BRCA1							
Intron 7	c.442-34 C > T (IVS7-34 C > T)	Polym.	No	ND	NA	2	DHPLC+Sequencing
Intron 7	c.442-18 C > T (IVS7-18 C > T)	VUS	ND	ND	NA	5	HRM+Sequencing
Exon 11	c.1067 A > G (p.Q356R)	Neutral	VUS	Conf. data** ^1^	C0	4	HRM+Sequencing
Exon 11	c.2082 C > T (p.S694S)	Neutral	VUS	B/LB	NA	8	Direct Sequencing
						7	DHPLC+Sequencing
Exon 11	c.2311 T > C (p.L771L)	Neutral	No	B/LB	NA	8	Direct Sequencing
						2	DHPLC+Sequencing
Exon 11	c.2612 C > T (p.P871L)	Neutral	No	B/LB	C0	7	Direct Sequencing
						8	DHPLC+Sequencing
Exon 11	c.3113 A > G (p.E1038G)	Neutral	No	B/LB	C0	9	Direct Sequencing
						5	DHPLC+Sequencing
Exon 11	c.3119 G > A (p.S1040N)	Neutral	VUS	Conf. data** ^2^	C0	2	Direct Sequencing
Exon 11	c.3548 A > G (p.K1183R)	Neutral	No	B/LB	C0	9	Direct Sequencing
						7	DHPLC+Sequencing
Exon 13	c.4308 T > C (p.S1436S)	Neutral	No	B/LB	NA	10	Direct Sequencing
Exon 16	c.4837A > G (p.S1613G)	Neutral	No	B/LB	C0	10	DHPLC+Sequencing
Intron 18	c.5152+66 G > A (IVS18+66 G > A)	Neutral	No	ND	NA	13	DHPLC+Sequencing
BRCA2							
5′UTR	c.-26G > A	Neutral	No	B/LB	NA	9	DHPLC+Sequencing
Intron 4	c.426-89T > C (IVS4-89T > C)	Neutral	No	VUS	NA	10	DHPLC+Sequencing
Intron 4	c.425+67A > C (IVS6+67A > C)	Neutral	No	VUS	NA	7	HRM+Sequencing
Intron 6	c.516+14C > T (IVS6+14C > T)	Lik. Neut.	ND	B/LB	NA	1	HRM+Sequencing
Exon 10	c.865A > C (p.N289H)	Neutral	No	B/LB	C0	3	HRM+Sequencing
						5	DHPLC+Sequencing
Exon 10	c.1114A > C (p.H372N)	Neutral	No	Conf. data** ^3^	C0	6	HRM+Sequencing
						14	HRM
Exon 10	c.1365A > G (p.S455S)	Neutral	No	B/LB	NA	8	HRM+Sequencing
						1	HRM
Exon 10	**c.1402A > G (p.R468G)**	ND	ND	ND	C0	1	HRM+Sequencing
						3	HRM
Intron 10	c.1910-74T > C (IVS10-74T > C)	Polym.	No	ND	NA	9	DHPLC+Sequencing
Exon 11	c.2229T > C (p.H743H)	Neutral	No	B/LB	NA	4	Direct sequencing
						1	DHPLC+Sequencing
Exon 11	c.2803G > A (p.D935N)	Neutral	No	B/LB	C0	1	HRM+Sequencing
Exon 11	**c.2842G > A (p.V948I)**	ND	ND	ND	C0	1	HRM+Sequencing
Exon 11	c.2971A > G (p.N991D)	Neutral	No	B/LB	C0	6	HRM+Sequencing
Exon 11	c.3396A > G (p.K1132K)	Neutral	No	B/LB	NA	20	Direct sequencing
Exon 11	c.3807T > C (p.V1269V)	Neutral	No	B/LB	NA	11	HRM+Sequencing
Exon 11	c.5096A > G (p.D1699G)	ND	VUS	VUS	C0	1	HRM+Sequencing
Exon 11	c.5199C > T (p.S1733S)	Neutral	No	B/LB	NA	1	HRM+Sequencing
Exon 11	c.5418A > G (p.E1806E)	Lik. Neut.	No	B/LB	NA	1	HRM+Sequencing
Exon 11	c.5744C > T (p.T1915M)	Neutral	No	Conf. data** ^4^	C0	3	HRM+Sequencing
Exon 11	c.6323G > A (p.R2108H)	Neutral	VUS	Conf. data** ^5^	C0	1	HRM+Sequencing
Intron 13	c.7008-62A > G (IVS13-62A > G)	Neutral	VUS	Conf.data** ^6^	NA	1	Direct sequencing
Exon 14	**c.7017G > A (p.K2339K)**	ND	ND	ND	NA	1	Direct sequencing
Exon 14	c.7242A > G (p.S2414S)	Neutral	No	B/LB	NA	10	HRM+Sequencing
Intron 16	c.7806-14T > C (IVS16-14T > C)	Neutral	VUS	B/LB	NA	15	DHPLC+Sequencing
Exon 18	c.8171G > T (p.G2724V)	ND	VUS	ND	C15	6	DHPLC+Sequencing
Exon 20	c.8567A > G (p.E2856A)	ND	No	ND	C0	2	DHPLC+Sequencing
Exon 22	c.8850G > T (p.K2950N)	Neutral	VUS	Conf. data** ^7^	C35	1	HRM+Sequencing
Exon 22	c.8851G > A (p.A2951T)	Neutral	No	B/LB	C0	3	HRM+Sequencing
Exon 23	c.9004G > A (p.E3002K)	VUS	VUS	Conf. data** ^8^	C55	1	HRM+Sequencing
Exon 26	c.9581C > A (p.P3194Q)	VUS	VUS	Conf. data**7	C0	1	HRM+Sequencing
Exon 27	c.10234A > G (p.I3412V)	Neutral	No	Conf. data**8	C0	2	HRM+Sequencing

* Nomenclature following HGVS recommendations

** Conf. data = Conflicting data from submitter (evaluated in July 2014). The superscript numbers correspond to the number between brackets. The number between parentheses means how many registries were made in each category. **[1]**: Benign (6), Likely benign (1), Uncertain significance (1); **[2]**: Benign (6), Likely benign (1), Pathogenic (1), Uncertain significance (1); **[3]** Benign (3), Pathogenic (1); **[4]** Benign (6), Likely benign (1), Uncertain significance (1); **[5]** Benign (4), Likely benign (1), Uncertain significance (1); **[6]** Benign (2), Uncertain significance (1); **[7]** Benign (2), Uncertain significance (1); **[8]** Likely pathogenic (1), Pathogenic (1) Uncertain significance (1); **[9]** Benign (1), Likeli benign (1), Uncertain significance (2); **[10]** Benign (5), Uncertain significance (1).

*** Align-GVGD score combines the biophysical characteristics of amino acids and protein multiple sequence alignments to predict where missense substitutions fall in a spectrum from enriched deleterious (C65, most likely to interfere with function) to enriched neutral (C0, least likely).

**Bold variants** highlight the new ones described in this study.

**B/BL** = Benign/Likely benign; **Lik. Net.** = Likely neutral; **NA** = Not applicable; **ND** = Not described; **Polym.** = Polymorphism; **VUS** = Variant of unknown significance;

#### LFL

One hundred twenty two families fulfilled the criteria for LFL syndrome (13.5% of the entire initial sample), being 13 and 119 families fulfilling Birch and Eeles criteria, respectively. Ten families had both Birch and Eeles criteria for LFL. Of the 122 families, 40 probands (from 40 unrelated families) underwent genetic testing Twelve of these 40 families (30%) also fulfilled criteria for at least on other BCPS (Table 2).

No deleterious mutation was found among the sequenced individuals, and the polymorphisms detected are shown in Table 4.

**Table 4:** *TP53* sequence variants identified in the 40 families fulfilling LFL syndrome criteria.

Localization	rs number	Alteration	ClinVar	Number of affected families
Intron 2	rs1642785	c.74+38C > G (IVS2+38C > G, PIN2)	Benign/Likely benign	30
Intron 3	rs17878362	c.96+25_96+40ACCTGGAGGGCTGGG (IVS3+24insACCTGGAGGGCTGGGG, PIN3)	Benign/Likely benign	16
Exon 4	rs1800370	c.108G > A (p.P36P)	Benign/Likely benign	2
Exon 4	rs1042522	c.215CG (p.P72R, PEX4)	Conflicting data*	34
Intron 7	rs12951053	c.782+92T > G (IVS7+92T > G)	ND	3
Intron 9	rs1800899	c.993+12T > C (IVS9+12T > C)	Benign/Likely benign	1

* Conflicting data = Conflicting data from submitter (evaluated in July 2014). The number between parentheses means how many registries were made in each category: Benign(4); Uncertain significance (1).

#### HBCC

**Figure 2:**
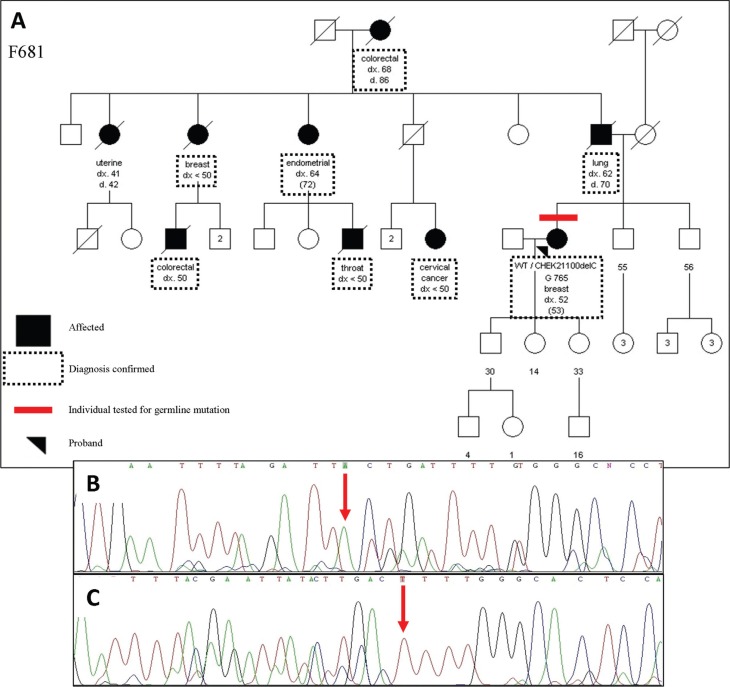
*CHEK2* 1100delC mutation family. Pedigree (A), forward (B) and reverse (C) sequencing of germline DNA. WT=wild type; dx=age at diagnosis; d=age of death; red arrows indicate the last readable base.

Among the 183 families with a hereditary breast cancer phenotype, 22 fulfilled criteria for HBCC (Meijers-Heijboer *et al.*, 2003), but only seven of them underwent genetic testing. Interestingly, all of the seven families also fulfilled criteria for a BCPS other than HBCC at inclusion in the study. The common *CHEK2* 1100delC mutation was identified in one of these seven families (14.3%) with multiple breast cancer diagnoses (proband had multiple breast cancers, first at age 52), colorectal cancer (ages 50 and 68 years), lung cancer and endometrial cancer (two cases) (Figure 2).

## Discussion

The identification and characterization of genetic alterations in families at high risk for breast cancer predisposition syndromes enables carriers to undertake individualized cancer screening and prevention strategies, thus increasing the likelihood of increased disease-free survival rates.

For HBOC, although we aimed at selecting patients at a somewhat higher prior probability of mutation than in most studies (average mutation probability between 20-30% using different criteria and prediction models) we identified a known deleterious germline mutation in only one of the 18 families studied. Using a strategy of screening for four common alterations in *BRCA1* and *BRCA2* (185delAG, 5382insC and exon 13 6kb duplication in *BRCA1* and 6174delT in *BRCA2*), Gomes (Gomes *et al.*, 2007) showed a mutation frequency of 2.3% of the 5382insC mutation in 402 Brazilian breast cancer patients unselected for family history. Although the average prior probability of mutation in their sample is not clear, one would expect that it was likely less (unselected sample) than the cutoff probability used for offering genetic testing in our report. In another Brazilian study that assessed *BRCA1* mutation prevalence in a group of 47 women from Rio de Janeiro, Lourenço *et al.* (Lourenço JJ VF, 2004), using more strict inclusion criteria, found 7 (15.0%) mutation carriers; again, 5382insC was one of the most common mutations encountered (4/7). Analyzing germline mutations in all coding regions of *BRCA1* and in common founder mutations in the *BRCA2*, *CHEK2* and *TP53* genes in a cohort of 106 high-risk HBOC patients, Felix *et al.* (Felix *et al.*, 2014) found 10 mutation carriers, and of them 9 harbored mutations in *BRCA1* (and none in *BRCA2*), performing a mutation frequency of 8,49%. A recent study of our group accessed the prevalence of three founder mutations (*BRCA1* c.68_69del, *BRCA1* c.5266dupC - former named as 5382insC, and *BRCA2* c.5946del) commonly identified in Ashkenazi individuals in a sample of 137 non-Ashkenazi cancer-affected women from Rio de Janeiro and Porto Alegre (all of them fulfilled clinical criteria for HBOC). The only mutation found was *BRCA1* c.5266dupC (5382insC), present in 7/137 women, a prevalence of 5.10% (Ewald *et al.*, 2011). A posterior study also conducted in Porto Alegre evaluated the prevalence of three Ashkenazi founder mutations (*BRCA1* 185delAG, *BRCA1* 5382insC and *BRCA2* 6174delT) in a group of 255 Ashkenazi Jewish women, non selected for personal or familial cancer history, and found a carrier frequency of 2/255 for 185delAG (0,78%), 1/255 for 6174delT (0,4%) and no mutated alleles for 5382insC, reveling a carrier frequency lower than expected for this ethnic group (Dillenburg *et al.*, 2012).

Indeed, *BRCA* mutation prevalence is probably heterogeneous, not only depending on criteria adopted for inclusion in a given study, but also related to testing methodology and specific features of different populations. For example, in a Finnish study of 128 HBOC patients, (Laurila *et al.*, 2005) did not identify any *BRCA1* mutation after sequencing the entire coding region of the gene. Finally, the effects of a small sample size and less than optimal confirmation of the cancer diagnoses in many of the families in our cohort must also be considered.

The negative findings of gene rearrangements in *BRCA1* and *BRCA2* could also be related to small sample size and, again, to the large variability of rearrangement prevalence. One complicating factor is that the knowledge about prevalence of such rearrangements in South American HBOC patients is limited and studies in other populations report a highly variable prevalence of such rearrangements. A Brazilian study conducted with 120 women fulfilling criteria for HBOC and screened for mutations, CNVs and rearrangements in *BRCA* and other genes found rearrangements of *BRCA1* in only two cases (exon 24 amplification and exon 16-17 deletion) (Silva *et al.*, 2014). In the Dutch, for example, large genomic rearrangements constitute 36% of the mutations detected in *BRCA1* (Petrij-Bosch *et al.*, 1997).

In spite of the large number of sequence variants identified in the *BRCA1/BRCA2* genes worldwide, many of them are still classified as VUS, and the available databases diverge about classification of variants. In this study, we found four *BRCA1* variants classified as VUS in at least one database, and two *BRCA2* variants that were consistently classified as VUS in all three analyzed databases, although one of them is, indeed, pathogenic (E3002K) (Biswas *et al.*, 2012; Cote *et al.*, 2012; Karchin *et al.*, 2008). Although the results are conflicting between the databases, functional studies done by Biswas *et al.* (2012) point to the fact that the mutation p.E3002K negatively impacts ssDNA binding and function, resulting in a deleterious phenotype. The findings on the likely pathogenicity of this mutation were corroborated by work published by Cote *et al.* (2012) in a recent study of 58 French Canadian families with breast and/or ovarian cancer and 960 cases not selected for family history of cancer, which found this variant in seven of the 58 families with a family history of cancer and in none of those not selected for family history. Additionally the AlignGVGD score for pathogenicity for this specific mutation is C55, pointing to a possibly/probably pathogenic alteration. Three novel sequence variants were found in *BRCA2*, two of them in the same individual. Further characterization of these novel variants is imperative and is underway. All efforts will be made to further characterize variants of unknown significance, especially if they were not described previously.

The finding of a high number of pedigrees suggestive of the LFL phenotype in our sample was somewhat surprising. First, because the questionnaire used to screen women from the general population for GCRA, was originally designed to identify the HBOC phenotype, and second because Li-Fraumeni syndrome and its variants have not been considered common cancer predisposition syndromes in most countries. However, Eeles I criteria, the only ones present in the majority of the 40 probands studied, are very relaxed and mutation prevalence in these families has been described as low (under 10%), which is in agreement with the absence of deleterious *TP53* mutations identified in our study. Interestingly, in other published mutation study of Brazilian LFL families (including 10 families from the State of Rio Grande do Sul), the mutation prevalence for LFL Eeles 1 and Birch families was much higher (23.1% and 61.5%, respectively). Again, our findings may be related to relatively small sample sizes and to the presence of lower-risk LFL families, some of them not having a sufficient number of cancer diagnoses confirmed to allow certainty about the BCPS phenotype. Finally, screening for the common *CHEK2-*1100delC mutation in seven HBCC families resulted in the identification of one mutation-positive family corresponding to a mutation prevalence of about 14%, (comparable to the 18% previously described in the literature) (Petrij-Bosch *et al.*, 1997). With the small sample size analyzed, these results could merely be related to chance, however they go along with other previous observations of a higher than expected number of colon cancers in the families breast-cancer affected women undergoing GCRA (Palmero *et al.*, 2009).

Two major limitations can be identified in our study. First, the relatively small sample size, which is not entirely unexpected. The limited acceptance of genetic testing that we faced throughout this study may be explained by cultural aspects of the population, low literacy of the majority of women counseled and this in turn could be associated with limited understanding of the benefits and implications of testing. Study design may also have influenced genetic testing acceptance. The women included in this study were recruited from their primary health care units and were not originally concerned about their genetic risk for cancer; or, if they were concerned, they did not seek genetic counseling directly. Furthermore, since our cohort was originated from a population-based sample, some of our probands were cancer-unaffected individuals from at-risk families for whom genetic testing required participation of at least one willing and alive cancer-affected relative. Another potential concern is that only a proportion of cancers in probands and/or relatives were confirmed, and therefore, there may be misclassifications of cancer site, tumor type as well as age at tumor diagnosis. Confirmation of cancer diagnoses in relatives, often distant ones, is usually challenging and in this population it may be particularly difficult, since many of them have lost contact with their families. All of these factors together may also contribute to poor understanding about increased risk and the existence and benefits of risk reduction strategies and consequent reduced motivation for genetic testing. In spite of the use of relatively strict inclusion criteria for testing of some BCPS (such as HBOC) and less strict criteria for others (LFL) and the comprehensive testing methodology used, the high risk posed to most of the families described here remains unexplained. Even considering the limitations highlighted before, this study raises several questions about the importance of genetic factors in determining the high breast cancer incidence and mortality rates in Southern Brazil and the acceptability of genetic testing in this population. In order to validate the low prevalence of germline mutations found in this work, a larger cohort should be analyzed. If indeed it is, we may have to revise criteria for genetic testing in this population and thoroughly investigate the contribution of different and/or novel high penetrance genes or the influence of multiple, more prevalent genetic variants of lower penetrance.

## Supplementary Material

The following online material mis available for this article: -

Table S1 - The Family History Syndrome questionnaire

This material is available as part of the online version of this article from http://www.scielo.br/gmb.
