# Erratum

**DOI:** 10.1590/1678-4685-GMB-2014-0363er

**Published:** 2017-03-16

In the article “Screening for germline BRCA1, BRCA2, TP53 and CHEK2 mutations in families at-risk for hereditary breast cancer identified in a population-based study from Southern Brazil”, with DOI number: http://dx.doi.org/10.1590/1678-4685-GMB-2014-0363, published in the journal Genetics and Molecular Biology, 39(2):210-222, on page 210 the author name spelled as Florence Le Calvez Kelm should read Florence Le Calvez-Kelm.

