# Supplementary material for: Screening for germline BRCA1, BRCA2, TP53 and CHEK2 mutations in families at-risk for hereditary breast cancer identified in a population-based study from Southern Brazil
Source: Genet Mol Biol. 2016 May 24;39(2):210–22. doi: 10.1590/1678-4685-GMB-2014-0363 (PMC4910552; doi:10.1590/1678-4685-GMB-2014-0363)
Supplement: Supplementary file 1 [file 1415-4757-gmb-1678-4685-GMB-2014-0363-Suppl01.pdf]

Table S1 - The Family History Syndrome questionnaire

1 Did any of your 1st degree relatives have breast *or* ovarian cancer?

2 Did any of your relatives have bilateral breast cancer?

3 Did any man in your family have breast cancer?

4 Did any woman in your family have breast *and* ovarian cancer?

5 Did any woman in your family have breast cancer before the age of 50 years?

6 Do you have 2 or more relatives with breast *and/or* ovarian cancer?

7 Do you have 2 or more relatives with breast *and/or* bowel cancer?
